# Assessment of Inflammation and Calcification in Pseudoxanthoma Elasticum Arteries and Skin with 18F-FluroDeoxyGlucose and 18F-Sodium Fluoride Positron Emission Tomography/Computed Tomography Imaging: The GOCAPXE Trial

**DOI:** 10.3390/jcm9113448

**Published:** 2020-10-27

**Authors:** Loukman Omarjee, Pierre-Jean Mention, Anne Janin, Gilles Kauffenstein, Estelle Le Pabic, Olivier Meilhac, Simon Blanchard, Nastassia Navasiolava, Georges Leftheriotis, Olivier Couturier, Pascale Jeannin, Franck Lacoeuille, Ludovic Martin

**Affiliations:** 1 Vascular Medicine Department, French National Health and Medical Research (Inserm), Clinical Investigation Center (CIC) 1414, University of Rennes 1, 35033 Rennes, France; 2 Pseudoxanthoma Elasticum (PXE) Clinical and Research Vascular Center, CHU Rennes, 35033 Rennes, France; 3 NuMeCan Institute, Exogenous and Endogenous Stress and Pathological Responses in Hepato-Gastrointestinal Diseases (EXPRES) team, French national health and medical research (Inserm) U1241, University of Rennes 1, 35033 Rennes, France; 4 Department of Nuclear Medicine, Angers University Hospital, 49100 Angers, France; PierreJean.Mention@chu-angers.fr (P.-J.M.); ocouturier70@me.com (O.C.); FrLacoeuille@chu-angers.fr (F.L.); 5 Sorbonne University Paris Nord, INSERM, U942, Cardiovascular Markers in Stressed Conditions, MASCOT, F- 93000 Bobigny, France; anne_janin@yahoo.com; 6 MitoVasc Institute Mixed Research Unit: National Centre for Scientific Research, CNRS 6015, French National Health and Medical Research, Inserm U1083, Angers University, 49100 Angers, France; gilles.kauffenstein@gmail.com (G.K.); Nastassia.Navasiolava@chu-angers.fr (N.N.); LuMartin@chu-angers.fr (L.M.); 7 CHU Rennes, French National Health and Medical Research (Inserm), Clinical Investigation Center (CIC) 1414, 35000 Rennes, France; Estelle.LE.PABIC@chu-rennes.fr; 8 University of Reunion Island, INSERM, UMR 1188 Reunion, Indian Ocean diabetic atherothrombosis therapies (DéTROI), CHU de La Réunion, 97400 Saint-Denis de La Réunion, France; olivier.meilhac@inserm.fr; 9 Regional Center for Research in Cancerology and Immunology Nantes/Angers, CRCINA, Angers University, 49100 Angers, France; simon.blanchard@univ-angers.fr (S.B.); Pascale.Jeannin@univ-angers.fr (P.J.); 10 Immunology and Allergology Department, CHU Angers, Angers University, 49100 Angers, France; 11 PXE Reference Center (MAGEC Nord), University Hospital of Angers, 49100 Angers, France; 12 Physiology and Vascular Investigation Department, CHU Nice, 06000 Nice, France; leftheriotis.g@chu-nice.fr; 13 GLIAD Team (Design and Application of Innovative Local Treatments in Glioblastoma), INSERM UMR 1232, CRCINA, CEDEX 9, 49933 Angers, France

**Keywords:** pseudoxanthoma elasticum, PET/CT, 18F-FDG, 18F-NaF, calcium score, arterial stiffness

## Abstract

Background: Pseudoxanthoma elasticum (PXE) is an inherited metabolic disease characterized by elastic fiber fragmentation and ectopic calcification. There is growing evidence that vascular calcification is associated with inflammatory status and is enhanced by inflammatory cytokines. Since PXE has never been considered as an inflammatory condition, no incidence of chronic inflammation leading to calcification in PXE has been reported and should be investigated. In atherosclerosis and aortic stenosis, positron emission tomography combined with computed tomographic (PET-CT) imaging has demonstrated a correlation between inflammation and calcification. The purpose of this study was to assess skin/artery inflammation and calcification in PXE patients. Methods: 18F-FluroDeoxyGlucose (18F-FDG) and 18F-Sodium Fluoride (18F-NaF) PET-CT, CT-imaging and Pulse wave velocity (PWV) were used to determine skin/vascular inflammation, tissue calcification, arterial calcium score (CS) and stiffness, respectively. In addition, inorganic pyrophosphate, high-sensitive C-reactive protein and cytokines plasma levels were monitored. Results: In 23 PXE patients, assessment of inflammation revealed significant 18F-FDG uptake in diseased skin areas contrary to normal regions, and exclusively in the proximal aorta contrary to the popliteal arteries. There was no correlation between 18F-FDG uptake and PWV in the aortic wall. Assessment of calcification demonstrated significant 18F-NaF uptake in diseased skin regions and in the proximal aorta and femoral arteries. 18F-NaF wall uptake correlated with CS in the femoral arteries, and aortic wall PWV. Multivariate analysis indicated that aortic wall 18F-NaF uptake is associated with diastolic blood pressure. There was no significant correlation between 18F-FDG and 18F-NaF uptake in any of the artery walls. Conclusion: In the present cross-sectional study, inflammation and calcification were not correlated. PXE would appear to more closely resemble a chronic disease model of ectopic calcification than an inflammatory condition. To assess early ectopic calcification in PXE patients, 18F-NaF-PET-CT may be more relevant than CT imaging. It potentially constitutes a biomarker for disease-modifying anti-calcifying drug assessment in PXE.

## 1. Introduction

Pseudoxanthoma elasticum (PXE, OMIM 264800) is a rare disorder characterized by fragmentation and progressive calcification of elastic fibers in connective tissue of the skin, vascular system and Bruch’s membrane of the retina [1]. PXE is caused by mutations in the *ABCC6* gene, encoding a transmembrane ATP-binding cassette (ABC) transporter primarily expressed in the liver and kidney [2,3]. ABCC6 endogenous substrates are as yet unknown. It was recently discovered that absence of ABCC6-mediated adenosine triphosphate release from the liver, causing reduced plasma inorganic pyrophosphate (PPi) levels, underlies calcification-induced PXE [4,5]. Peripheral artery disease (PAD) resulting from calcification in the internal elastic lamina of the medial layer in muscular and elastic arteries is highly prevalent in PXE patients and mimics vascular calcification observed in other acquired metabolic diseases such as diabetes mellitus (DM) and chronic kidney disease (CKD) [6,7]. Vascular calcification is the result of a regulated process that is orchestrated by vascular smooth muscle cells (VSMCs) and develops similarly to the physiological mineralization process [6,7]. In normal vessels, VSMCs are protected from calcification by mineralization inhibitors such as PPi and matrix Gla protein [4,5,7]. However, VSMCs can die by apoptosis in response to an attack, releasing apoptotic bodies that contain calcium phosphate crystals as identified in atherosclerotic lesions and medial vascular calcification (MVC) [7]. Calcium phosphate crystals have been shown in vitro to induce a pro-inflammatory response involving cytokine release (IL-1β, IL-6, TNFα) in cultures of differentiated human macrophages that potentially leads to a vicious cycle of pro-inflammatory macrophage infiltration, extracellular matrix breakdown and VSMC apoptosis [8]. There is growing evidence that vascular calcification is connected to inflammatory status and is enhanced by inflammatory cytokines [7]. Studies have suggested that in DM-related PAD, atherosclerosis or CKD, low grade chronic inflammation (LGCI) may occur prior to vascular calcification (VC) due to immune-cell recruitment and pro/anti-inflammatory cytokine imbalance [6,7]. Since PXE has never been considered as an inflammatory condition, no incidence of chronic inflammation leading to calcification in PXE has been reported and as such requires investigation. Non-invasive techniques such as positron emission tomography combined with computed tomographic imaging (PET-CT) imaging have demonstrated a correlation between inflammation and calcification in atherosclerosis [9,10] and aortic stenosis [11]. 18F-FluroDeoxyGlucose (18F-FDG) and 18F-sodium fluoride (18F-NaF), PET tracers for inflammation and active mineral deposition respectively, have been used in several dual tracer PET-CT studies designed to investigate vascular conditions where LGCI and VC, also called inflammaging [12], are deemed key pathogenic factors [13]. The purpose of the present study was to investigate skin/artery LGCI using 18F-FDG-PET-CT, and tissue calcification using 18F-NaF-PET-CT. Additionally, artery wall 18F-NaF-PET-CT activity and arterial calcium scores obtained from CT-scans were compared. We also investigated any correlation between: 18F-FDG-PET-CT/18F-NaF-PET-CT and PWV; 18F-FDG-PET-CT and hsCRP; 18F-NaF-PET-CT and PPi plasma levels. An attempt was made to determine evidence of a distinctive circulating blood factor such as cytokines in PXE patients.

## 2. Experimental Section

### 2.1. Clinical Trial Registration

This GOCAPXE study was registered under reference number NCT 03070860. https://clinicaltrials.gov/ct2/show/NCT03070860?term=NCT03070860&draw=2&rank=1.

### 2.2. Patients

#### 2.2.1. Ethical Standards

The data supporting the findings herein are available upon reasonable request from the corresponding author. The said author accepts responsibility for the reliability of all study data to which full access was provided, including data analysis.

The trial protocol was approved by the local research ethics committee (CPP Ouest II, Angers, France; EudraCT identification number: 2014-A01614-43 and CPP identification number 2014/35) and was implemented as per the most recent amendments to the Declaration of Helsinki and good clinical practice guidelines. Written informed consent was obtained from all patients prior to enrolment. The GOCAPXE study was registered with Clinicaltrials.gov on 6 March 2017 (NCT 03070860) and has been overseen by an independent data safety and monitoring committee. No control group of patients undergoing PET-CT imaging was included in this trial for obvious ethical reasons related to radiation risks. The lead author wrote the first manuscript draft, and each co-author contributed to and validated subsequent revised versions.

#### 2.2.2. Patient Population

From 2017–2018, PXE patients and healthy volunteers (HVs) were enrolled prospectively in the present trial at the National Reference Center for PXE at Angers University Hospital. Following written informed consent, each participant was examined for screening purposes. During this examination detailed medical history was obtained including drug use, smoking habits, and family medical background. Each patient received a comprehensive baseline clinical examination including evaluation of each cardiovascular risk factor profile.

#### 2.2.3. PXE Patients

PXE diagnosis was established genetically and/or clinically in accordance with international diagnostic criteria for clear-cut PXE: (i) evidence of angioid streaks on eye funduscopy; (ii) typical skin lesions featuring yellowish papules or large coalescent plaques in the neck/flexural region; (iii) skin biopsy demonstrating dermal elastorrhexis and calcification by positive von Kossa staining [14]. *ABCC6* mutations were identified through genotyping (Supplementary Material Table S1).

Exclusion criteria were: women of childbearing age using no contraception; pregnant or breastfeeding women; diabetic patients; patients with osteopenia, inflammatory or autoimmune systemic disease; patients with high blood glucose (>11 mmol/L) due to potential competition between glucose and 18F-FDG. Each patient received a comprehensive baseline clinical examination, including evaluation of each cardiovascular risk factor profile.

#### 2.2.4. Healthy Volunteers

HVs were recruited prospectively by the Clinical Investigation Center of Angers University Hospital and matched to PXE patients by age and sex.

### 2.3. Clinical and Biological Assessment

Data collected from both PXE patients and HVs included: age; sex; BMI; AHT; smoking habits; DM; dyslipidemia; family/personal medical history of cardiovascular disease (MI); stroke; lower extremity PAD. ABI was measured according to existing guidelines [15]. PAD was defined as ABI ≤ 0.90 or >1.40 [15]. Laboratory blood analysis from PXE patients and HVs was compared. Total LDL/HDL-cholesterol, triglyceride and blood-glucose levels were measured from fasting blood samples using standard laboratory techniques. CVR factors were defined as follows: (1) AHT: SBP > 140 mmHg and DBP > 90 mmHg and/or antihypertensive medication; (2) DM: fasting plasma glucose ≥ 7.0 mmol/L (≥126 mg/dL) and/or random plasma glucose ≥ 11.1 mmol/L (≥200 mg/dL) and/or HbA1c ≥ 6.5% (≥48 mmol/L), and/or glucose-lowering medication; (3) dyslipidemia: LDL-cholesterol > 3.4 mmol/L, HDL-cholesterol <1 mmol/L, triglyceridemia > 2 mmol/L and/or lipid-lowering medication.

Blood testing involved: hsCRP mg/L using standard laboratory techniques; PPi (µmol/L) [5] assay; several immunological tests. Each blood sample was drawn following an 8-hour overnight fasting period.

#### 2.3.1. Multiplex Immunoassays Using Luminex^®^ Technology

Associations of change in functional assessment were investigated using biological assays of a set of circulating blood factors. Plasma samples from 23 PXE patients and 23 HVs were obtained from the Angers University Hospital BRC. 46 chemokines, cytokines, growth factors, lectin adhesion molecules, osteogenic factors, matrix metalloproteinase and fibrogenic factors were quantified in PXE patient and HV sera using Luminex assay kits following manufacturer instructions (R&D Systems). Samples were analyzed using a Luminex 200 analyzer and Bio-Plex Manager version 6 software. The following substances were analyzed: CCL2, CCL3, CCL4, CCL5, CCL17, CCL18, CCL22, CXCL10, IFNγ, IL-1Ra, IL-1β, IL-4, IL-6, IL-8, IL-10, IL-12p70, IL-17a, TNFα, TGF β, G-CSF, M-CSF, GM-CSF, VEGF, HGF, PDGF-BB, E-selectin, P-selectin, L-selectin, BMP-2, BMP-4, OA, OPN, ON, fetuin-A, OPG, RANKL, MMP-1, MMP-2, MMP-3, MMP-7, MMP-8, MMP-9, MMP-10, MMP-12, endothelin and PAI-1.

#### 2.3.2. PPi Assay

For PXE patients only, plasma PPi was measured by enzymatic reaction using ATP sulfurylase to convert PPi into ATP in the presence of excess adenosine-5′-phosphosulfate (Sigma-Aldrich, St. Louis, MO, USA), as previously described [5].

### 2.4. Aortic Stiffness Assessment

#### Carotid–Femoral PWV Measurement

Carotid–femoral PWV resulting from aortic stiffness was recorded tonometrically. Transcutaneous carotid–femoral PWV in the right common carotid and femoral arteries was recorded consecutively on a high-resolution tonometer (PulsePen, DiaTecne, Milan, Italy) [16]. ECG signals were used as a time reference. Distance (in mm) between the two recording sites was measured with a ruler and calculated as direct carotid–femoral distance corrected by a factor equal to 0.8 as recommended by the European Society of Hypertension [17]. Carotid–femoral PWV was determined by an intersecting tangent algorithm and expressed in mm/s.

### 2.5. Vascular and Skin Imaging

#### 2.5.1. PET-CT and CT-Scan Imaging Techniques

PXE patients underwent all-body 18F-FDG/18F-NaF-PET-CT and CT-scans. Spared regions such as the popliteal arteries (vascular investigation) [1] or the lumbar region (skin investigation) were regarded as negative controls in these patients.

Patients fasted for at least 6 hours prior to intravenous 225 ± 56 MBq (3 MBq/kg) injection of 18F-FDG. Data were acquired on a dedicated PET-CT Discovery-690 system (LYSO scintillation PET detector; 16-slice CT; GE^®^, Buc, France) with an acquisition time of 2 min/bed position. The PET images were reconstructed using: an OSEM 3D algorithm (3 iterations, 8 subsets, FOV 700 mm, 192 × 192 matrix, 3.65 mm pixels, 3.27 mm slice thickness, post-reconstruction Gaussian 4 mm filter); a VPFX TOF algorithm; PSF correction (Sharp IR). CT-based attenuation correction (120 kV, Auto mA, 20 mm collimation, 1.375 pitch, 0.8 s/rot) was applied.

Two days later, patients were injected with 218 ± 57 MBq (3 MBq/kg) 18F-NaF using a dedicated PET-CT Discovery ST system (BGO scintillation PET detector; 8-slice CT; GE, Buc, France) at acquisition times of 3 min/bed position (head/thorax/pelvis) and 2 min/bed position (legs). The PET images were reconstructed using an OSEM 3D algorithm (2 iterations; 30 subsets, FOV 500 mm, 128 × 128 matrix, 3.9 mm pixels, 3.27 mm slice thickness, post reconstruction standard 4.29 mm filter). CT-based attenuation correction (120 kV, 80 mA, 20 mm collimation, 1.35 pitch, 0.8 s/rot) was applied.

Whole-body PET-CT images were obtained within 60 min of 18F-FDG injection, and 90 min of 18F-NaF injection.

Data on calcified plaque burden (ALCS) were acquired from Philips 64 Brilliance CT-scans (120 kV, auto mA, 40 mm collimation, 1 mm pitch, 0.5 s/rot, 1.5 mm slice thickness).

#### 2.5.2. Image Analysis: 18F-FDG/18F-NaF-PET-CT

**Figure 1:**
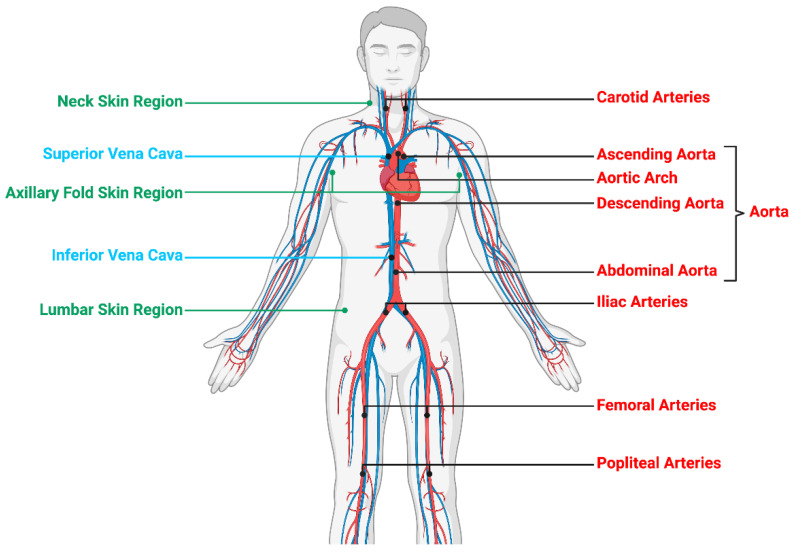
**Whole-body 18F-FDG/18F-NaF-PET/CT to assess subclinical arterial inflammation and active mineral deposition**. Skin/arterial inflammation quantified as 18F-FDG SUVmax. Active mineral deposition quantified as 18F-NaF SUVmax. SUVmax was determined by manually drawing an individual ROI 1cm^3^ around the most fixed arterial segment. TBRmax in vascular system obtained by dividing artery SUVmax by vena cava (blood pool) SUVmax. SUVmax measured from ascending to abdominal aorta as mean total aorta (meanTBR) in addition to neck, axillary fold and lumbar skin regions.

Each image dataset was analyzed by a nuclear medicine physician on an Imagys^®^ workstation (Keosys^®^, Saint-Herblain, France). Maximum intensity projection PET-CT images were assessed visually for evidence of radiotracer accumulation in the femoral or popliteal artery walls, as previously described [18]. In semi-quantitative analysis, maximum SUV was determined by manually drawing an individual ROI 1 cm^3^ around the most fixed arterial segment (Figure 1) that included: the left and right carotid arteries, aorta (ascending aorta, aortic arch, descending aorta, abdominal aorta), left and right iliac arteries, damaged left and right femoral arteries and spared left and right popliteal arteries, as shown on coregistered transaxial PET-CT findings. The ROI was adjusted to the vascular wall using coronal and sagittal PET-CT images. Blood pool SUVmax/mean was expressed as the SUVmax/mean of a 1 cm fixed diameter ROI drawn mid lumen in the superior and inferior vena cava, as previously described [19]. The SUVmax of each artery lesion was divided by blood pool SUVmax to ascertain TBRmax [13,20]. For the purposes of analysis, mean TBR in the left and right carotid, iliac, femoral, popliteal arteries and the proximal (ascending aorta and aortic arch) and distal aorta (descending and abdominal aorta) was calculated [18] (Figure 1). TBR in the left and right carotids and the proximal aorta was calculated by dividing the SUVmax by SUVmax derived from the superior vena cava [21]. TBR in the distal aorta, iliac, femoral and popliteal arteries was calculated by dividing the SUVmax by SUVmax derived from the inferior vena cava [21]. With respect to skin analysis, SUVmax and SUVmean were measured after a circular ROI had been drawn around three regions (spared lumbar/damaged neck/axillary folds) [22]. Mean linear SUV was calculated in the left and right axillary folds [22].

#### 2.5.3. Image Analysis: CS in Lower Limb Arteries

Each PXE patient was submitted to a non-contrast-enhanced-64-row-multidetector-CT scan (Brillance 64, Philips HealthCare, Dest, The Netherlands) of the lower limbs, from the iliac crest to the tips of the toes, without injection of contrast medium [23].

Investigators blinded to patient clinical status calculated CS using automated 3D image-analysis software (Synapse 3D, Fujifilm Medical Systems, Greenwood, SC, USA). ROIs were divided into 3 segments for each individual leg: (1) the femoral segment (common and femoral arteries), extending from the iliac crest to the adductor magnus opening; (2) the popliteal segment (from adductor magnus opening to origin of anterior tibial artery); (3) the sub-popliteal segment (anterior and posterior tibialis and fibular arteries from their origin to malleolar region) [23]. Each segment length was measured using 3D scans and expressed in mm. Calcified regions with a cross-sectional area ≥0.7 mm^2^ and density ranging between 150–400 HU (Hounsfield Unit) were automatically identified on cross-sectional lower-extremity images. The CS was ascertained and expressed as ALCS in each segment of both legs [24]. The ALCS of each segment was normalized to its length (arbitrary units). Arteries with CS = 0HU were regarded as non-calcified.

### 2.6. Statistical Analysis

Continuous variables were expressed as mean ± SD/median and IQR values. Categorical variables were expressed as counts/percentages. The Student’s *t*-test (or Mann-Whitney Wilcoxon exact test where appropriate) was used to compare continuous variables and the chi-squared test (or Fisher’s exact test where appropriate) to compare categorical variables.

The Wilcoxon signed-rank test was used to analyze LGCI on 18F-FDG-PET-CT and ectopic calcification on 18F-NaF-PET-CT.

Univariate analysis using linear regression or variance analysis was applied to investigate factors associated with artery wall 18F-FDG/18F-NaF uptake. Variables with *p* < 0.20 were then selected for multivariate analysis. The dependent variable was artery wall 18F-FDG/18F-NaF uptake (mean TBR), and independent covariates were selected on the assumption they were linked to 18F-FDG/18F-NaF uptake and CVR factors (PWV, SBP, DBP, right/left ABI, age, sex, BMI, HbA1c, smoking, total cholesterol, LDL, hsCRP, PPi).

Backward stepwise analysis was applied. Normal distribution of the measured variables was verified.

The Spearman coefficient was used to analyze correlation. A statistical significance threshold of 0.05 was adopted for all tests. SAS^®^ 9.4 software (SAS Institute, Cary, NC, USA) was used for statistical analysis.

## 3. Results

### 3.1. Patient Population

23 patients (aged 47 ± 14; 52% female) were subjected to whole body 18F-NaF-PET-CT and 18F-FDG-PET-CT three days apart. Concurrently, 23 HVs were recruited (aged 46 ± 13; 52% female) to compare blood samples. Clinical characteristics of the study population are shown in Table 1.

### 3.2. Assessment of LGCI

**Figure 2:**
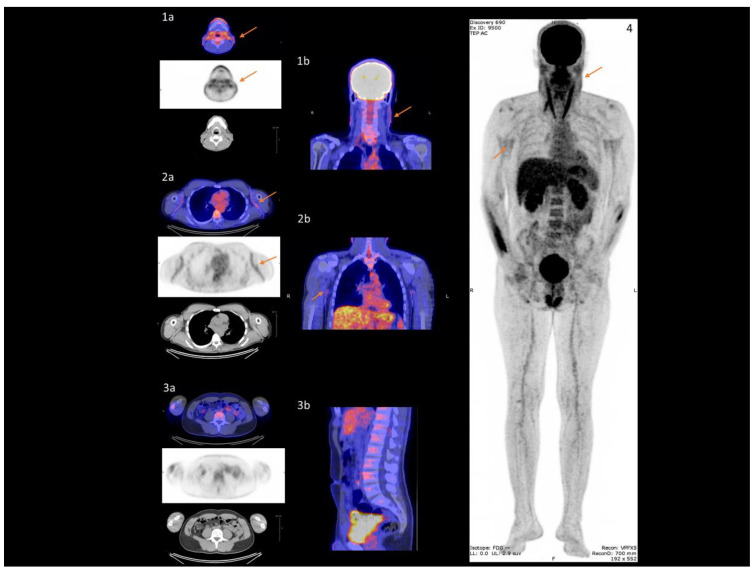
**18F-FDG uptake in specific damaged skin in PXE**. Damaged skin in neck (arrows (**1a**,**1b**)) and axillary folds (arrows (**2a**,**2b**)) vs healthy skin in lumbar region (**3a**,**3b**) on PET/CT imaging. Maximum intensity projection indicates damaged skin sites (arrow 4).

In all 23 PXE patients, skin imaging revealed significantly higher 18F-FDG uptake in the neck (Figure 2(1a,1b)) and axillary folds (Figure 2(2a,2b)) than in normal lumbar skin (Figure 2(3a,3b)) (neck SUV = 1.30 (1.20;1.90) or axillary folds SUV = 1.80 (1.60;1.90) versus lumbar SUV = 0.90 (0.80;1.10); *p* < 0.0001), (Table 2). On vascular imaging, no difference in 18F-FDG uptake was found between the carotid, distal aorta, iliac, and popliteal artery wall. By contrast, there was evidence of significantly higher 18F-FDG uptake in the proximal aorta and femoral than in the popliteal artery wall (proximal aorta TBR = 1.32 (1.13;1.57) versus popliteal TBR = 0.91 (0.80;1.14); *p* < 0.0001), (femoral TBR = 0.89 (0.75;1.02) versus popliteal TBR = 0.91 (0.80;1.14); *p* = 0.03), (Table 2). No correlation was found between PWV and aorta wall 18F-FDG uptake (Supplementary Material, Figure S1a). Multivariate analysis established a significant link between BMI/HbA1c and aorta wall 18F-FDG uptake (*p* = 0.01 and *p* = 0.02 respectively).

In the subgroup of 11 PXE patients with CS = 0HU, no difference was found in 18F-FDG uptake between the carotid, distal aorta, iliac, femoral and popliteal artery wall. By contrast, 18F-FDG uptake was significantly higher in the proximal aorta than in the popliteal artery wall (proximal aorta TBR = 1.24 (1.05;1.43) versus popliteal TBR = 1.06 (0.73;1.14); *p* = 0.03) (Table 2). No correlation was found between PWV and aorta wall 18F-FDG uptake (Supplementary Material, Figure S1b). Multivariate analysis demonstrated no significant link between aorta wall 18F-FDG uptake and covariates.

18F-FDG uptake in all arteries walls (see Figure 1) was not correlated with hsCRP levels (Supplementary Material, Figure S1c).

### 3.3. Assessment of Ectopic Calcification

**Figure 3:**
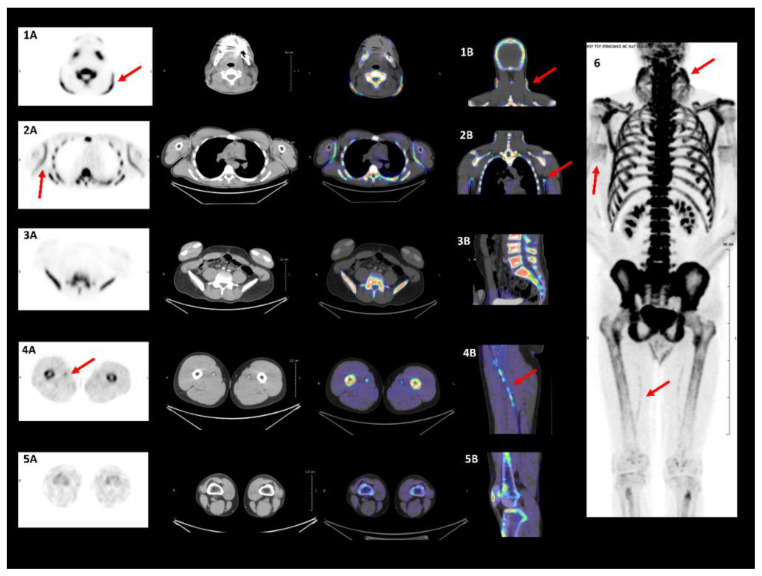
**18F-NaF uptake in specific damaged skin/arteries in PXE**. Damaged skin in neck (arrows (**1A**,**1B**)) and axillary folds (arrows (**2A**,**2B**)) vs healthy skin in lumbar region (**3A**,**3B**) on PET/CT imaging. Molecular calcification targeted with 18F-NaF. Damaged femoral arteries on PET/CT imaging. Molecular calcification targeted via PET/CT demonstrating 18F-NaF femoral arteries wall uptake (arrows (**4A**,**4B**)). 18F-NaF uptake absent in popliteal arteries walls (**5A**,**5B**). Maximum intensity projection indicates damaged vascular and skin sites (arrow 6).

**Figure 4:**
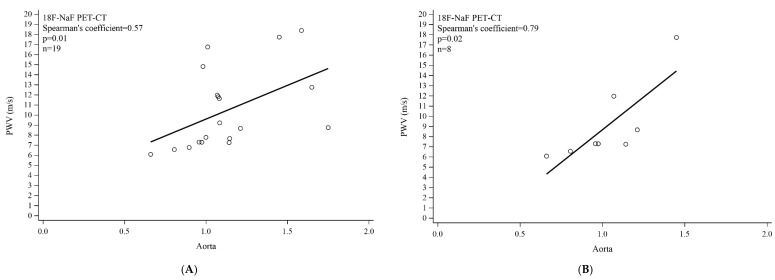
Correlation between 18F-NaF uptake and pulse wave velocity (PWW) in aorta. (**A**) All PXE patients; (**B**) PXE patients with calcium score (CS) = 0HU.

On skin imaging of all 23 PXE patients, 18F-NaF uptake was significantly higher in the neck (Figure 3(1A,1B)) and axillary folds (Figure 3(2A,2B)) than in the lumbar skin region (Figure 3(3A,3B)): (neck SUV = 4.50 (3.20;5.10) or axillary folds SUV = 2.75 (1.90;3.30) versus lumbar SUV = 0.70 (0.50;0.90); *p* < 0.0001) (Table 2). Vascular imaging showed comparable 18F-NaF uptake in the carotid, distal aorta, iliac, and popliteal artery wall. 18F-NaF uptake was significantly higher in the proximal aorta and femoral than in the popliteal artery wall (proximal aorta TBR = 1.42 (1.25;1.63) versus popliteal TBR = 1.12 (0.99;1.30); *p* = 0.0004), (femoral TBR = 1.55 (1.26;2.00) versus popliteal TBR = 1.12 (0.99;1.30); *p* < 0.0001) (Figure 3(4A,4B,5A,5B)), (Table 2). Significant correlation was found between PWV and aorta wall 18F-NaF uptake in all PXE patients (Spearman’s coefficient = 0.57; *p* = 0.01) (Figure 4A). This was equally the case after adjustment for SBP, DBP, and both together (Table 3) Multivariate analysis demonstrated a significant link between DBP and aorta wall 18F-NaF uptake (*p* = 0.01).

In the subgroup of 11 PXE patients with CS = 0HU (*n* = 11), no difference in 18F-NaF uptake was found between the distal aorta, iliac and popliteal artery walls. By contrast, there was evidence of significantly higher 18F-NaF uptake in the carotid, proximal aorta and femoral artery walls than in that of the popliteal artery (carotid TBR = 1.14 (0.93;1.92) versus popliteal TBR = 1.00 (0.85;1.15); *p* = 0.01), (proximal aorta TBR = 1.29 (1.06;1.64) versus popliteal TBR = 1.00 (0.85;1.15); *p* = 0.003), (femoral TBR = 1.50 (1.11;1.79) versus popliteal TBR = 1.00 (0.85;1.15); *p* = 0.02), (Table 2). Significant correlation was found between PWV and aortic wall 18F-NaF uptake (*n* = 8) (Spearman’s coefficient = 0.79; *p* = 0.02) (Figure 4B). Multivariate analysis showed a significant link between smoking and aortic wall 18F-NaF uptake (*p* = 0.01).

No correlation was established between 18F-NaF uptake in any of the artery walls (see Figure 2) and plasma PPi levels (Supplementary Material, Figure S1d).

Significant correlation was observed between femoral artery-wall 18F-NaF uptake and CS (Spearman’s coefficient = 0.54; *p* = 0.01).

### 3.4. 18F-FDG/18F-NaF Uptake Correlation in the Vascular Network

**Figure 5:**
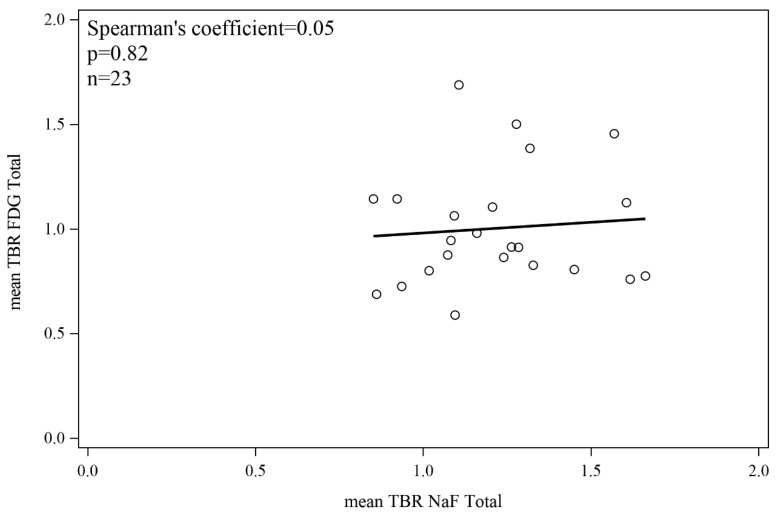
Correlation between 18F-FDG and 18F-NaF uptake in artery walls.

No significant correlation was detected between 18F-FDG and 18F-NaF uptake in any of the artery walls (Figure 5).

### 3.5. Assessment of Blood Circulating Factors

**Figure 6:**
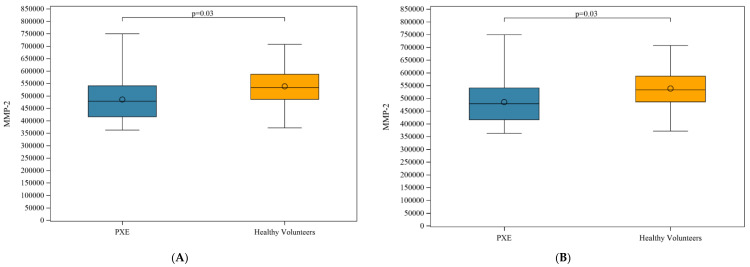
(**A**) Plasma levels of MMP-2 in PXE patients and healthy volunteers; (**B**) MMP-3 in PXE patients and healthy volunteers.

A significant difference was observed in MMP-2 and MMP-3 plasma levels between PXE patients and HVs (Figure 6A,B).

No difference was observed in plasma levels of chemokines, cytokines, growth factors, lectin adhesion molecules, osteogenic factors or fibrogenic factors between PXE patients and HVs (Table 4).

## 4. Discussion

The present study has demonstrated that 18F-FDG/18F-NaF activity was significantly greater in PXE-damaged skin regions and in the proximal aorta wall, whereas 18F-NaF activity alone was greater in the femoral arteries.

### 4.1. PXE: A Seemingly Non-Inflammatory Condition

We have previously demonstrated that specific skin regions (neck/flexural regions) and arteries (aorta/femoral/leg) are affected by PXE lesions while the lumbar skin region and popliteal arteries are spared [1,23]. Owing to the high levels of 18F-FDG activity observed in specific regions, the question arises as to whether LGCI is involved. No histological study has so far provided evidence of inflammatory cells in skin biopsies obtained from PXE patients [25]. Higher 18F-FDG uptake in specific skin regions may reflect pathological fibroblast proliferation in PXE [26]. Similarly, histological and ultrastructural analysis [27] of PXE artery walls has failed to detect inflammatory or immune cells such as those found in vasculitis [28] or atherosclerosis [7]. Moreover, no immune-inflammatory pathway in PXE patients was identified by the Luminex study, unlike in Takayasu arteritis where Th1 and Th17 cytokines drive inflammation [28].

An increase in ascending-aorta 18F-FDG activity is the only factor giving credence to early LGCI in PXE patients. Using multivariate analysis, we have shown that this 18F-FDG uptake appears to be significantly linked to BMI and HbA1c as commonly observed in DM [21]. These factors may therefore contribute to a LGCI state in PXE patients.

Furthermore, recent studies on the ascending aorta in atherosclerosis have demonstrated that VSMCs and fibroblasts, acting as macrophages, are capable of accumulating 18F-FDG [29,30]. They conclude that ascending-aorta 18F-FDG activity does not necessarily signify inflammation and advise against regarding this region as an imaging endpoint [30,31].

Taken together, our findings imply no LGCI in PXE.

### 4.2. PXE as a Prime Example of Chronic Skin and Arterial Calcification

In the 23 PXE patients 18F-NaF activity was higher in the neck and axillary folds than in the lumbar skin region, suggesting active calcification on damaged skin. These results are consistent with those recently published where patients with higher skin Phenodex scores exhibited higher 18F-NaF uptake in the neck [32]. In the vascular system 18F-NaF uptake was observed exclusively in the aorta and the femoral arteries, substantiating our previous characterization of vulnerability to PXE damage in these regions [1,23,33]. Similarly, MVC and non-calcified atherosclerotic lesions constitute a predominant leg artery calcification type in the general population. In a series of 121 leg amputees, MVC was found in 71% of femoral and crural arteries versus 25% of calcified atherosclerotic lesions [34]. MVC is, moreover, a strong predictor for major cardiovascular events [35].

We found a trend for inverse correlation between 18F-NaF and PPi plasma levels. It is likely that the correlation was not significant due to lack of power. This observation did however lead us to hypothesize about the kinetics of ectopic calcification. As 18F-NaF activity increases [33], PPi rates decrease [4,5]. Nevertheless, it is possible that the decrease in PPi plasma levels alone does not account for ectopic calcification [36].

We also detected a decrease in MMP-2 and MMP-3 plasma levels in PXE patients. By contrast, elevated levels of circulating MMP-2 and MMP-9 reflecting extracellular matrix remodeling have been found in the sera of German PXE patients [37]. The assays were conducted on serum [37] and not on plasma. Serum is obtained after coagulation that results in thrombus formation, causing the release of high amounts of MMP-9 through neutrophil degranulation [38]. In addition, MMP-2 was found to be elevated exclusively in the sera of women in the German PXE cohort [37]. MMP3 degrades fibronectin, proteoglycans, laminin, basal lamina collagen IV, and collagen telopeptides. It enhances MMP-1 collagenolytic activity by enhancing fibrillar collagen hydrolysis [39]. A decrease in MMP3 may favor collagen accumulation, causing fibrosis as a result. Ectopic calcification conceivably leads to fewer MMP-2- and MMP-3-producing cells as in atherosclerosis, whereby calcification often corresponds to areas containing either no cells or dead cells [40].

Taken together, PXE is conceivably a prime example of chronic skin and arterial calcification.

### 4.3. Aortic Stiffness Correlated with 18F-NaF Not 18F-FDG in PXE

In 44 early-onset DM patients, aortic stiffness correlated with 18F-FDG [21]. In the 23 PXE cases studied herein, aortic stiffness correlated with 18F-NaF irrespective of SBP, DBP or both together but not with 18F-FDG. Two previous studies have shown that calcification in the tunica media of PXE patients increases arterial stiffness [41,42]. Arterial stiffness and MVC have been shown to correlate [43,44] and to be independent predictors of cardiovascular morbidity and mortality [45,46]. In the present study, multivariate analysis revealed that the adjusted risk factors for aortic MVC were DBP in all patients, and tobacco use in the subgroup with CS = 0HU. Nicotine can induce osteogenic transdifferentiation in VSMCs [47] resulting in tunica media calcification of the vessel wall [47].

Bartstra et al., evoked PXE as a prime example of accelerated peripheral vascular aging whereby MVC induces CV disease independently of atherosclerosis, inflammation and thrombosis [35]. In the present work, 18F-NaF activity in the vascular system has tended to correlate inversely with PPi levels. PPi is the major calcification inhibitor lacking in PXE patient plasma [5], and loss of a single calcification inhibitor can initiate MVC [4,48,49].

### 4.4. 18F-NaF as a Diagnostic and Follow-Up Biomarker in PXE

18F-NaF-PET-CT is able to identify calcification that cannot be detected by CT-resolution alone [50]. It is recognized as a reliable detector for quantifying ectopic calcification [33] and tracking its progression [50].

In addition, linear femoral artery 18F-NaF uptake correlates with CVR factors [51].

We have demonstrated herein that 18F-NaF-PET-CT is able to detect early-onset calcification in patients with CS = 0HU. 18F-NaF is therefore a biomarker candidate for the diagnosis and follow-up of cardiovascular disease in PXE.

In the “Treatment of Ectopic Mineralization in Pseudoxanthoma Elasticum (TEMP)” trial, etidronate, a non-nitrogen-containing bisphosphonate and a PPi analog, reduced arterial calcification on CT-Scan but did not lower femoral 18F-NaF activity [52]. In this study, etidronate was administered similarly to treatment of osteoporosis (cyclical 20 mg/kg for two weeks every 12 weeks) [52]. In PXE, calcification is a slow and continuous process [27]. Discontinuous administration of etidronate in PXE is effective on clinically visualized CT calcification [52] but may not be effective at reducing molecular calcification as assessed by 18F-NaF. Since PPi is the main anti-calcifying agent that is lacking in PXE patients [4,5], we can hypothesize that discontinuous administration of PPI or its analogs could promote the restarting of molecular calcifications. This may explain the lack of 18F-NaF decay in the femoral arteries in PXE patients treated discontinuously with etidronate [52]. A clinical trial evaluating continuous versus discontinuous administration of etidronate might answer this question by retaining as primary endpoint 18F-NaF quantification of molecular calcification in the femoral arteries.

### 4.5. Study Limitations

The present study lacked a control group for dual PET-CT imaging since each patient was his/her own control for ethical reasons. Additionally, dual PET-CT imaging was conducted on non-digital PET scanners.

## 5. Conclusions

In the present cross-sectional study (using FDG/plasma biomarkers), no link could be established between inflammation and calcification in PXE patients.

PXE would appear to more closely resemble a chronic disease model of ectopic calcification than an inflammatory condition. To assess early ectopic calcification in PXE patients, 18F-NaF-PET-CT may be more relevant than CT imaging. It potentially constitutes a biomarker for disease-modifying anti-calcifying drug assessment in PXE.

### Clinical Perspectives for PXE Patients

Should 18F-NaF-PET-CT prove to be an early biomarker of vascular and skin calcification in PXE patients, it may constitute an endpoint when assessing disease-modifying anti-calcifying drugs in PXE. Physicians caring for PXE patients are advised to keep CVR factors under control to minimize arterial stiffness and MVC.

## Figures and Tables

**Table 1:** Baseline Population Characteristics.

Patient Characteristics	All	PXE Patients	Healthy Volunteers	*p*
Age	46	23	23	0.79
(mean ± sd)	46.67 ± 13.53	47.22 ± 14.10	46.13 ± 13.23	
Sex	46	23	23	1.00
Female	24 (52.2%)	12 (52.2%)	12 (52.2%)	
Male	22 (47.8%)	11 (47.8%)	11 (47.8%)	
BMI (Kg/m^2^)	44	23	21	0.20
(median (IQR))	23.57 (22.23;26.54)	24.01 (22.68;28.41)	23.24 (21.07;25.68)	
SBP (mmHg)	40	23	17	0.15
(median (IQR))	120.50 (112.50;131.50)	121.00 (114.00;142.00)	118.00 (110.00;126.00)	
DBP (mmHg)	40	23	17	0.60
(median (IQR))	70.00 (63.00;74.50)	69.00 (63.00;78.00)	71.00 (66.00;73.00)	
Right ABI	40	23	17	0.10
(mean ± sd)	1.02 ± 0.18	0.98 ± 0.20	1.07 ± 0.14	
Left ABI	40	23	17	0.16
(mean ± sd)	1.02 ± 0.20	0.98 ± 0.23	1.07 ± 0.13	
Total Cholesterol (mmol/L)	39	23	16	0.80
(mean ± sd)	5.20 ± 1.01	5.24 ± 0.95	5.16 ± 1.12	
LDL Cholesterol (mmol/L)	38	23	15	0.84
(mean ± sd)	3.21 ± 0.87	3.23 ± 0.89	3.17 ± 0.86	
HbA1c %	31	20	11	0.76
(mean ± sd)	5.44 ± 0.45	5.42 ± 0.51	5.47 ± 0.33	
hsCRP (mg/L)	46	23	23	0.49
	1.74 ± 2.70	2.11 ± 3.45	1.37 ± 1.67	
PPi (μmol/L)	17	17		
Only for PXE patients	7.00E-07 ± 6.00E-07	7.00E-07 ± 6.00E-07		
Current Smoker	46	23	23	0.02
No	40 (87.0%)	17 (73.9%)	23 (100.0%)	
Yes	6 (13.0%)	6 (26.1%)	0 (0.0%)	
Stroke	46	23	23	1.00
No	45 (97.8%)	22 (95.7%)	23 (100.0%)	
Yes	1 (2.2%)	1 (4.3%)	0 (0.0%)	
Myocardial Infarction	46	23	23	1.00
No	45 (97.8%)	22 (95.7%)	23 (100.0%)	
Yes	1 (2.2%)	1 (4.3%)	0 (0.0%)	
PAD	46	23	23	0.002
No	37 (80.4%)	14 (60.9%)	23 (100.0%)	
Yes	9 (19.6%)	9 (39.1%)	0 (0.0%)	

BMI: Body Mass Index; ABI: Ankle-Brachial Index; SBP: Systolic Blood Pressure; DBP: Diastolic Blood Pressure; PAD: Peripheral Arterial Disease; sd: Standard Deviation; IQR: Interquartile Range; LDL: Low Density Cholesterol; HbA1c: Glycated Hemoglobin; PXE: Pseudoxanthoma Elasticum.

**Table 2:** Assessment of LGCI using 18F-FDG-PET-CT and ectopic calcification using 18F-NaF-PET-CT.

	**All PXE Patients (*n* = 23)**							
	**18F-FDG PET-CT**		**18F-NaF PET-CT**					
**SKIN**	SUV max	*p*	SUV max	*p*				
Lumbar (Reference Region) (median (IQR))	0.90 (0.80;1.10)		0.70 (0.50;0.90)					
Neck (median (IQR))	1.30 (1.20;1.90)	<0.0001	4.50 (3.20;5.10)	<0.0001				
Axillary Folds (median (IQR))	1.80 (1.60;1.90)	<0.0001	2.75 (1.90;3.30)	<0.0001				
	**All PXE Patients (*n* = 23)**				**PXE Patients with CS = 0HU (*n* = 11)**			
	**18F-FDG PET-CT**		**18F-NaF PET-CT**		**18F-FDG PET-CT**		**18F-NaF PET-CT**	
**ARTERIES**	TBR max	*p*	TBR max	*p*	TBR max	*p*	TBR max	*p*
Popliteal (Reference Region) (median (IQR))	0.91 (0.80;1.14)		1.12 (0.99;1.30)		1.06 (0.73;1.14)		1.00 (0.85;1.15)	
Carotid (median (IQR))	1.04 (0.88;1.18)	0.93	1.25 (0.93;1.75)	0.16	0.88 (0.74;1.08)	0.32	1.14 (0.93;1.92)	0.01
Aorta (Ascending and Arch) (median (IQR))	1.32 (1.13;1.57)	<0.0001	1.42 (1.25;1.63)	0.0004	1.24 (1.05;1.43)	0.03	1.29 (1.06;1.64)	<0.01
Aorta (Descending and Adominal) (median (IQR))	1.02 (0.88;1.36)	0.09	0.78 (0.70;1.04)	0.07	0.95 (0.88;1.29)	0.24	0.78 (0.70;0.90)	0.19
Iliac (median (IQR))	0.93 (0.74;1.15)	0.29	0.90 (0.75;1.32)	0.48	0.93 (0.63;1.04)	0.23	0.86 (0.65;1.00)	0.52
Femoral (median (IQR))	0.89 (0.75;1.02)	0.03	1.55 (1.26;2.00)	<0.0001	0.88 (0.79;1.02)	0.31	1.50 (1.11;1.79)	0.02

PXE: Pseudoxanthoma Elasticum; IQR: Interquartile Range; CS: Calcium Score; HU: Hounsfield Unit; PET-CT: Positron Emission Tomography combined with Computed Tomography; 18F; FDG: 18F-Flurodeoxyglucose; 18F-NaF: 18F-Sodium Fluorure; SUV: Standard Unit Value; TBR: Tissue-to-Blood pool Ratio.

**Table 3:** Correlation between aorta wall 18F-NaF uptake and PWV adjusted to SBP, DBP and both together.

PXE Patients (*n* = 19)		
Adjusted Correlation between Aorta Wall 18F-NaF Uptake and PWW (m/s)	Spearman Partial Correlation Coefficient	*p*
Adjusted correlation for SBP (mmHg)	0.57	0.01
Adjusted Correlation for DBP (mmHg)	0.57	0.01
Adjusted correlation for SBP (mmHg) and DBP (mmHg)	0.58	0.02

PWV: Pulse Wave Velocity; SBP: Systolic Blood Pressure; DBP: Diastolic Blood Pressure.

**Table 4:** Chemokines, cytokines, growth factors, lectin adhesion molecules, osteogenic factors, matrix metalloproteinase and fibrogenic factors in plasma samples from PXE patients and Healthy Volunteers.

	All (*n* = 46) Median (IQR)	PXE (*n* = 23) Median (IQR)	Healthy Volunteers (*n* = 23) Median (IQR)	*p*
**Chemokines**				
CCL2	264.69 (225.28;345.65	250.01 (229.88;333.75)	289.51 ± 104.64	0.66
CCL3	0.00 (0.00;56.43)	0.00 (0.00;82.68)	0.00 (0.00;56.43)	0.24
CCL4	0.00 (0.00;0.00)	0.00 (0.00;0.00)	0.00 (0.00;0.00)	0.49
CCL5	30,948 (23,723;38,964)	34,155 (23,041;38,964)	29,601 (25,421;39,548)	0.83
CCL17	392.21 (314.88;541.28)	364.38 (296.11;515.60)	429.37 (317.54;548.24)	0.40
CCL18	44,428 (30,810;59,277)	44,817 (30,863;54,648)	41,288 (25,807;60,200)	0.86
CCL22	504.85 (460.23;598.45)	521.49 (472.01;598.45)	495.82 (405.80;615.17)	0.14
CXCL10	21.73 (16.29;26.58)	22.74 (16.29;25.01)	20.42 (15.98;28.05)	0.98
**Cytokines**				
IL-1ra	587.78 (420.12;832.53)	591.79 (447.93;912.15)	497.71 (360.76;799.96)	0.18
IL-1 β	0.00 (0.00;0.00)	0.00 (0.00;0.00)	0.00 (0.00;0.00)	0.34
IL-4	17.38 (17.38;25.84)	25.84 (17.38;33.44)	17.38 (7.06;25.84)	0.22
IL-6	0.17 (0.00;0.62)	0.36 (0.00;0.75)	0.00 (0.00;0.36)	0.08
IL-8	7.23 (6.07;9.96)	7.62 (6.07;11.15)	6.84 (6.07;8.01)	0.24
IL-10	0.00 (0.00;0.00)	0.00 (0.00;0.00)	0.00 (0.00;0.00)	1.00
IL-12p70	0.00 (0.00;0.00)	0.00 (0.00;0.00)	0.00 (0.00;0.00)	1.00
IL-17A	0.00 (0.00;0.00)	0.00 (0.00;0.00)	0.00 (0.00;0.00)	0.34
IFNγ	2.50 (0.00;2.50)	2.50 (0.00;2.50)	0.49 (0.00;4.74)	0.77
TNFα	0.00 (0.00;0.00)	0.00 (0.00;0.00)	0.00 (0.00;0.00)	0.61
TGFβ1	112,133 (100,387;126,496)	112,509 (103,813;137,997)	110,248 (91,732;121,820)	0.16
**Growth Factors**				
G-CSF	0.00 (0.00;0.76)	0.00 (0.00;1.82)	0.00 (0.00;0.00)	0.71
M-CSF	0.00 (0.00;0.00)	0.00 (0.00;0.00)	0.00 (0.00;0.00)	0.95
GM-CSF	0.00 (0.00;0.00)	0.00 (0.00;0.00)	0.00 (0.00;0.00)	0.15
VEGF-A	44.46 (18.48;62.10)	47.49 (18.48;74.52)	38.02 (18.48;48.83)	0.25
HGF	132.26 (105.26;184.94)	169.00 (108.29;217.93)	131.51 (102.23;169.73)	0.20
PDGF-BB	5826.27 (4693.62;7024.87)	6323.78 (4767.44;7167.26)	5703.14 (4515.93;6796.59)	0.23
**Lectin Adhesion Molecules**				
E-Selectin	22,206.12 (17,767.67;34,471.30)	23,188.95 (17,204.99;35,267.87)	21,407.08 (17,767.67;30,674.42)	0.97
L-Selectin	600,330.64 (531,235.56;688,938.90)	610,241.04 (548,413.23;678,355.84)	598,640.51 (523,798.06;709,249.07)	0.95
P-Selectin	49,405.90 (37,883.69;61,905.20)	42,381.27 (35,790.69;61,905.20)	51,578.79 (41,755.65;65,608.85)	0.40
**Osteogenic Factors**				
BMP-2	58.56 (54.02;61.45)	58.56 (52.45;61.45)	58.56 (54.02;64.24)	0.67
BMP-4	44.49 (40.51;54.85)	44.49 (36.13;53.25)	46.36 (40.51;56.41)	0.40
Osteoactivin	22,685.20 (21,353.14;24,640.44)	22,504.34 (20,212.75;24,640.44)	22,890.26 (21,353.14;24,934.29)	0.91
Osteopontin	18,437.45 (14,947.65;21,517.65)	19,004.67 (15,037.00;21,670.99)	18,110.55 (12,618.80;21,517.65)	0.52
Osteonectin	2,588,600.00 (2,255,700.00;2,924,200.00)	2,820,200.00 (2,229,000.00;3,311,800.00)	2,545,400.00 (2,255,700.00;2,780,800.00)	0.15
Osteoprotegerin	628.63 (539.63;736.51)	658.26 (571.15;813.95)	584.38 (502.39;707.36)	0.09
RANKL	9.21 (6.37;11.16)	10.18 (8.25;11.16)	9.21 (5.44;11.16)	0.08
Fetuin-A	487,970,000 (422,850,000;601,000,000)	512,070,000 (440,520,000;601,000,000)	463,690,000 (413,500,000;602,390,000)	0.47
**Matrix Metalloproteinase**				
MMP-1	2021.21 (1534.73;3406.60)	2154.55 (1534.73;3862.47)	1915.57 (1300.14;3310.01)	0.48
MMP-2	514,982.65 (457,406.57;548,911.69)	478,598.33 (416,138.80;540,719.70)	533,325.91 (486,092.57;587,136.67)	0.03
MMP-3	9704.09 (6387.33;16,945.20)	7574.66 (5631.33;9816.17)	14,108.42 (8212.26;20,466.87)	0.02
MMP-7	9050.0 ± 1073.3	8928.1 ± 1169.9	9171.8 ± 978.05	0.45
MMP-8	5432.3 (4744.7;6715.9)	4977.4 (4468.4;6715.9)	5544.3 (4783.8;6715.9)	0.51
MMP-9	170,124.09 (135,202.28;237,051.11)	168,637.52 (135,202.28;224,464.92)	194,727.27 (132,832.22;285,870.08)	0.55
MMP-10	967.85 (813.53;1315.12)	925.02 (776.52;1132.34)	993.13 (813.53;1330.80)	0.45
MMP-12	34.91 (28.45;47.99)	31.89 (24.60;52.38)	37.92 (31.89;37.92)	0.79
**Fibrogenic Factors**				
Endothelin-1	0.00 (0.00;0.00)	0.00 (0.00;0.00)	0.00 (0.00;0.00)	0.34
PAI-1	91,900.85 (80,754.77;116,870.76)	100,095.18 (83,731.81;125,177.18)	88,542.09 (72,077.73;102,371.86)	0.13

PXE: Pseudoxanthoma Elasticum; CCL: Chemokine (C-C motif) Ligand; CXCL: Chemokine (C-X-C motif) Ligand; IL: Interleukin; INF: Interferon; TNF: Tumor Necrosis Factor; TGF: Transforming Growth Factor; G-CSF: Granulocyte Colony Stimulating Factor; M-CSF: Monocyte Colony Stimulating Factor; GM-CSF: Granulocyte Monocyte Colony Stimulating Factor; VEGF: Vascular Endothelial Growth Factor; HGF: Hepatocyte Growth Factor; PDGF: Platelet Dirived Growth Factor; BMP: Bone Morphogenic Protein; RANKL: Receptor Activator of Nuclear factor Kappa-B Ligand; MMP: Matrix Metalloproteinase; PAI: Plasminogen Activator Inhibitor; sd: Standard Deviation; IQR: Interquartile Range.

## Supplementary Materials

The following are available online at https://www.mdpi.com/2077-0383/9/11/3448/s1, Supplementary Table S1: *ABCC6* mutations in PXE patients; Figure S1: a,b: Correlation between 18F-FDG uptake and pulse wave velocity (PWW) in aorta; a: All PXE patients; b: PXE patients with calcium score (CS) = 0; c: Correlation between 18F-FDG uptake in all arteries walls and hsCRP, d: Correlation between 18F-NaF uptake in all arteries walls and PPi; Video 1: Typical 18F-NaF uptake in the femoral arteries of a Pseudoxanthoma Elasticum Patient.

## Abbreviations

18F-FDG: 18F-Flurodeoxyglucose; 18F-NaF: 18F-Sodium fluoride; ABCC6: ATP-binding cassette sub-family C member 6; ABI: Ankle-brachial index; AHT: Arterial hypertension; ALCS: Agatston-like calcium scores; ATP: Adenosine triphosphate; BMI: Body mass index; BMP-2: Bone morphogenetic protein-2; BRC: Biological resource center; CKD: Chronic kidney disease; CVR: Cardiovascular risk; CCL2: Monocyte chemoattractant protein 1 (MCP1), CCL3: Macrophage inflammatory protein 1-alpha (MIP-1-alpha); CCL4: Macrophage inflammatory protein-1β (MIP-1β); CCL5: Regulated on activation, normal T cell expressed and secreted (RANTES); CCL17: Thymus and activation regulated chemokine (TARC); CCL18: Macrophage inflammatory protein-4 (MIP-4); CCL22: Macrophage-derived chemokine, (MDC); CS: Calcium scoring; CXCL10: IFNγ–inducible 10-kDa protein (IP-10); DBP: Diastolic blood pressure; DM: Diabetes mellitus; ECG: Electrocardiogram; FL: Framingham-Laurier risk score; G-CSF: Granulocyte colony-stimulating factor; GM-CSF: Granulocyte–macrophage colony-stimulating factor; HbA1c: Hemoglobin A1c; HGF: Hepatocyte growth factor; hsCRP: High-sensitive C-reactive protein; HU: Hounsfield units; HVs: Healthy volunteers; IFNγ: Interferon gamma; IL: Interleukin; IL-1Ra: IL-1 receptor antagonist; IQR: Interquartile range; LDL: Low-density lipoprotein; LGCI: Low grade chronic inflammation; MBq: Megabecquerel; M-CSF: Macrophage colony-stimulating factor; MI: Myocardial infarction; MMP: Matrix metalloproteinase; MVC: Medial vascular calcification; OA: Osteoactivin; OMIM: Online Mendelian Inheritance in Man; ON: Osteonectin; OPG Osteoprotegerin; OPN: Osteopontin; OSEM: Ordered-subset expectation maximization; PAD: Peripheral artery disease; PAI-1: Plasminogen activator inhibitor-1; PDGF-BB: Platelet-derived growth factor BB; PET-CT: Positron emission tomography combined with computed tomography; PPi: Inorganic pyrophosphate; PSF: Point spread function; PWV: Pulse wave velocity; PXE: Pseudoxanthoma Elasticum; RANKL: Receptor activator of nuclear factor kappa-B ligand; ROI: Region of interest; SBP: Systolic blood pressure; SD: Standard deviation; SUV: Standard uptake value; TBR: Tissue-to-blood x ratio; TGF β: Transforming growth factor β; TNFα: Tumor necrosis factor α; TOF: Time-of-flight; VC: Vascular calcification; VEGF: Vascular endothelial growth factor.
