# Supplementary material for: Assessment of Inflammation and Calcification in Pseudoxanthoma Elasticum Arteries and Skin with 18F-FluroDeoxyGlucose and 18F-Sodium Fluoride Positron Emission Tomography/Computed Tomography Imaging: The GOCAPXE Trial
Source: J Clin Med. 2020 Oct 27;9(11):3448. doi: 10.3390/jcm9113448 (PMC7692997; doi:10.3390/jcm9113448)
Supplement: Supplementary file 1 [file jcm-09-03448-s001.zip › jcm-938075-supplementary (2)/jcm-938075-supplementary.docx]

**Supplementary Materials**

**Table S1: *ABCC6* mutations in PXE patients**

| **PXE Patient** | **Sexe** | **Age** | **Mutation 1** | **Mutation 2** |
| --- | --- | --- | --- | --- |
| 1 | Female | 65 | p.Arg1164Gln | p.Arg1164Gln |
| 2 | Male | 37 | p.Arg765Gln | p.Gln1406Lys |
| 3 | Female | 33 | p.Arg1141Ter | p.Arg1141Ter |
| 4 | Male | 65 | p.Arg1141Ter | p.Arg1141Ter |
| 5 | Male | 41 | p.Asp777Asn | p.Asp777Asn |
| 6 | Male | 73 | p.Arg1141Ter | p.Arg1357Trp |
| 7 | Female | 68 | p.Arg1141Ter | IVS8+2 delTG |
| 8 | Male | 57 | p.Arg1141Ter | del23_29 |
| 9 | Female | 51 | p.Arg1141Ter | p.Arg1141Ter |
| 10 | Female | 43 | p.Gln378Ter | p.Ala384Pro |
| 11 | Female | 45 | del 23_29 | del 23_29 |
| 12 | Male | 19 | IVS8+2delTG | p.Gly992Arg |
| 13 | Male | 55 | del9_10 | del9_10 |
| 14 | Female | 24 | p.Trp38Ser | p.Arg518Gln |
| 15 | Male | 56 | p.Arg1141Ter | p.Arg1314Gln |
| 16 | Female | 45 | p.Thr1130Met | p.Ala1303Pro |
| 17 | Male | 40 | p.Arg1141 Ter | p.Arg1141 Ter |
| 18 | Male | 51 | p.Arg1138Gln | p.Arg1030Ter |
| 19 | Female | 26 | del23_29 | c.1892-1943+26del28 |
| 20 | Female | 39 | del23_29 | p.Arg1141Ter |
| 21 | Male | 52 | p.Arg518Gln | p.Glu1400Lys |
| 22 | Female | 45 | p.Arg1141Ter | IVS21+1 G>T |
| 23 | Female | 56 | c.36+1G>C | c.1868-5T>G |

| 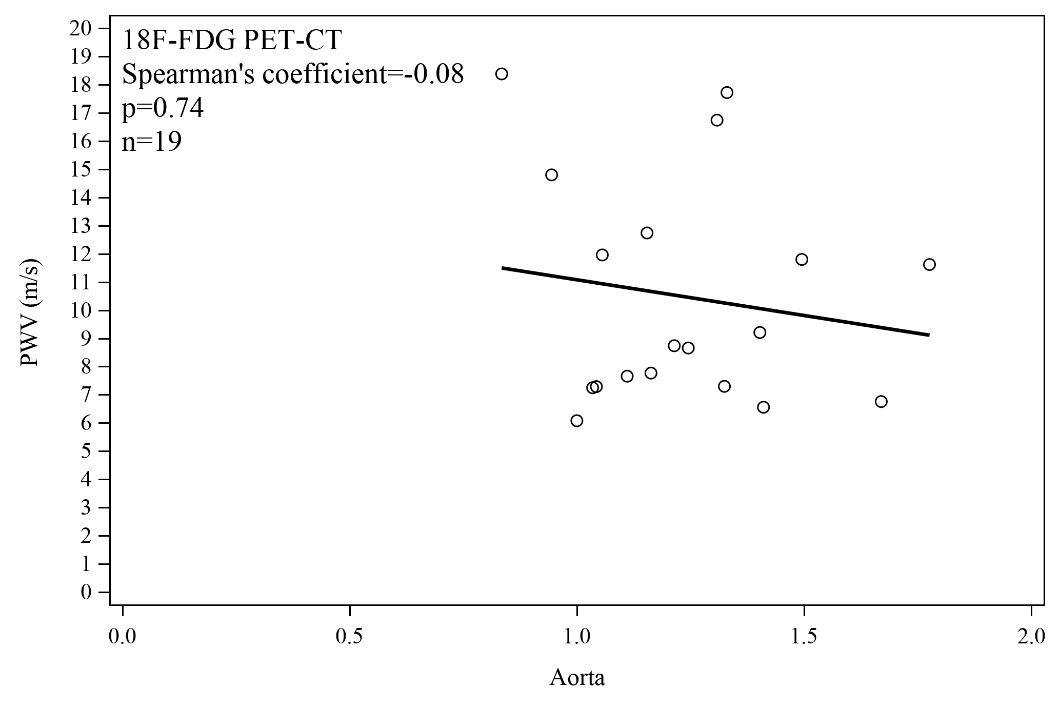 | 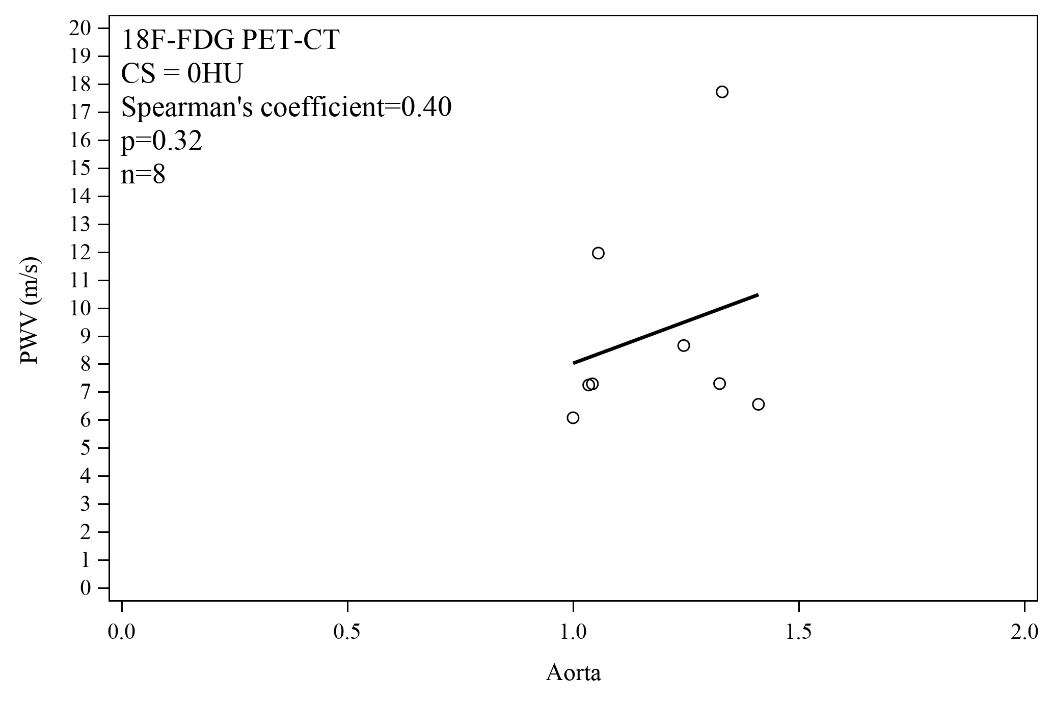 |
| --- | --- |
| **a** | **b** |
| 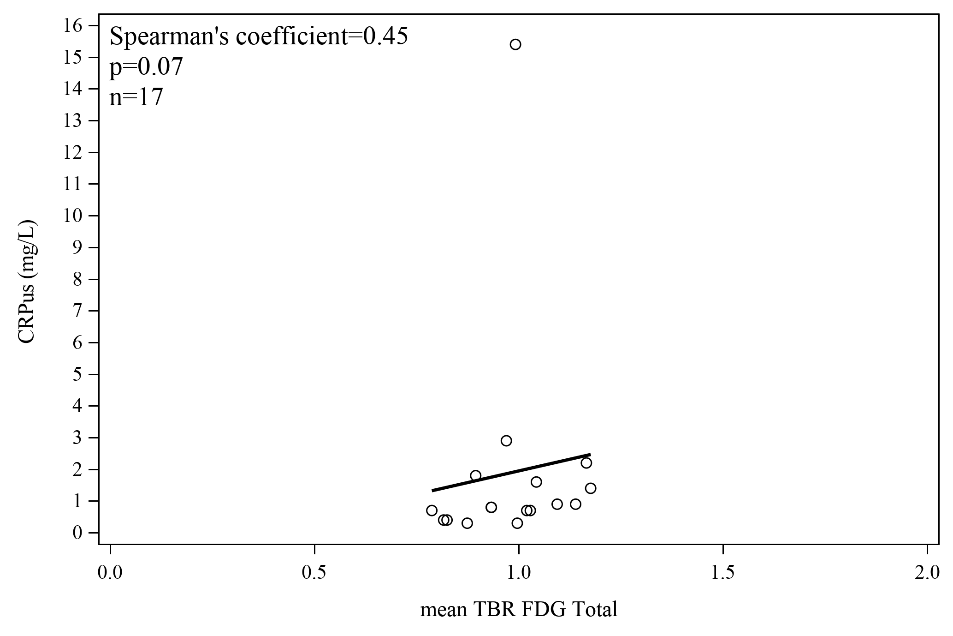 | 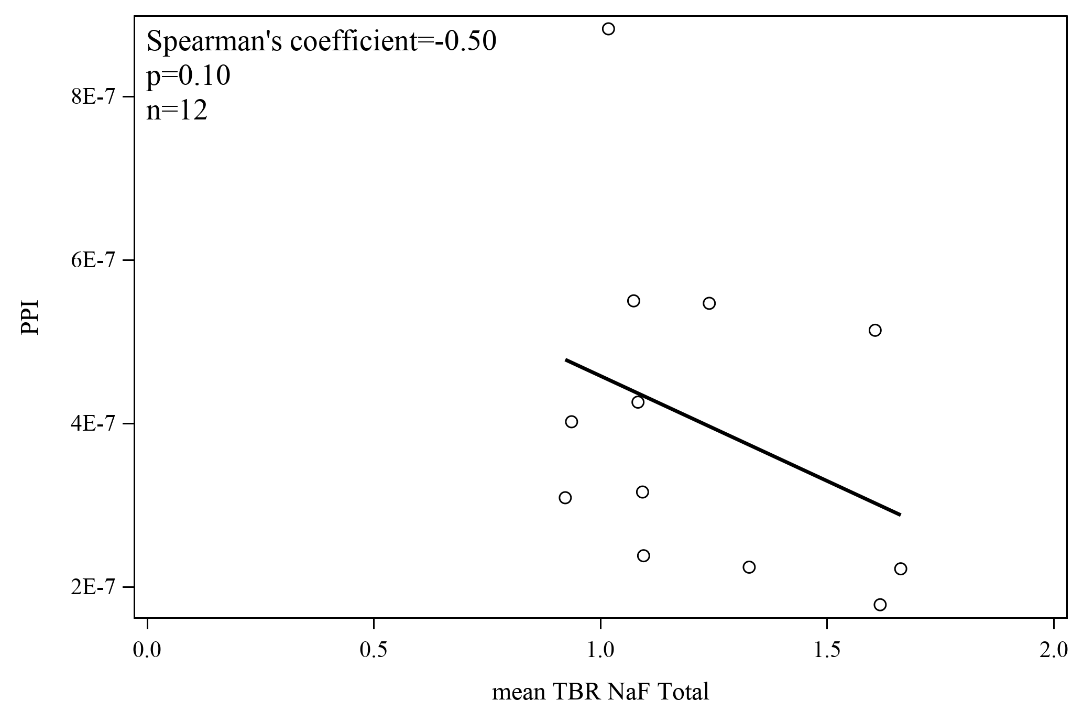 |
| **c** | **d** |

**Figure S1: a & b: Correlation between 18F-FDG uptake and pulse wave velocity (PWW) in aorta. a: All PXE patients; b: PXE patients with calcium score (CS) = 0**

**c: Correlation between 18F-FDG uptake in all arteries walls and hsCRP. d: Correlation between 18F-NaF uptake in all arteries walls and PPi.**
